# Optimizing Anesthetic Practices for Mud Crab: A Comparative Study of Clove Oil, MS-222, Ethanol, and Magnesium Chloride

**DOI:** 10.3390/antiox12122124

**Published:** 2023-12-16

**Authors:** Lulu Zhu, Shanshan Qi, Ce Shi, Shujian Chen, Yangfang Ye, Chunlin Wang, Changkao Mu, Ronghua Li, Qingyang Wu, Xiaopeng Wang, Yueyue Zhou

**Affiliations:** 1 Marine Economic Research Center, Donghai Academy, Ningbo University, Ningbo 315000, China; zhululu0426@163.com (L.Z.); qishanshan0616@163.com (S.Q.); chenshujian@nbu.edu.cn (S.C.); 2 Key Laboratory of Aquacultural Biotechnology, Ningbo University, Chinese Ministry of Education, Ningbo 315000, China; yeyangfang@nbu.edu.cn (Y.Y.); wangchunlin@nbu.edu.cn (C.W.); muchangkao@nbu.edu.cn (C.M.); lironghua@nbu.edu.cn (R.L.); wuqingyang@nbu.edu.cn (Q.W.); wangxiaopeng@nbu.edu.cn (X.W.); zhouyueyue@nbu.edu.cn (Y.Z.); 3 Key Laboratory of Green Mariculture (Co-Construction by Ministry and Province), Ministry of Agriculture and Rural, Ningbo 315000, China; 4 Collaborative Innovation Center for Zhejiang Marine High-Efficiency and Healthy Aquaculture, Ningbo 315000, China

**Keywords:** *Scylla paramamosain*, anesthetic, physiological response, antioxidant capacity, neurotransmitters, metabolomics

## Abstract

Anesthesia serves as an effective method to mitigate the stress response in aquatic animals during aquaculture and product transportation. In this study, we assessed the anesthetic efficacy of clove oil, tricaine methane-sulfonate (MS-222), ethanol, and magnesium chloride by anesthesia duration, recovery time, 24-hour survival rate, and the behavior of mud crabs (*Scylla paramamosain*). Additionally, the optimal anesthetic concentration for varying body weights of mud crabs was also investigated. The results revealed that clove oil emerged as the optimal anesthetic for mud crabs, with a 24-hour survival rate surpassing those observed in MS-222 and magnesium chloride treatments. Ethanol caused amputation and hyperactivity in mud crabs. Regression analyses between the optimal anesthetic concentration of clove oil and the weight categories of 0.03–27.50 g and 27.50–399.73 g for mud crabs yielded the following equations: y = 0.0036 x^3^ − 0.1629 x^2^ + 1.7314 x + 4.085 (R^2^ = 0.7115) and y = 0.0437 x + 2.9461 (R^2^ = 0.9549). Clove oil exhibited no significant impact on serum cortisol, glucose, lactate content, aspartate aminotransferase (AST), alanine aminotransferase (ALT) activities, or superoxide dismutase (SOD), catalase (CAT), glutathione peroxidase (GSH-Px), and malondialdehyde (MDA) levels in mud crabs across different treatment groups. Anesthesia induced by clove oil in mud crabs resulted in an increase in inhibitory neurotransmitters such as glycine. However, the recovery from anesthesia was associated with elevated levels of the excitatory neurotransmitters L-aspartic acid and glutamate. In conclusion, clove oil proves to be a safe and optimal anesthetic agent for mud crabs, exerting no physiological stress on the species.

## 1. Introduction

In aquaculture and the transportation of aquatic products, interventions such as measuring, vaccinating, handling, and classifying are essential for optimizing harvest outcomes. However, these processes can induce physical damage and physiological stress in aquatic animals, which increases susceptibility to pathogen infections and eventual mortality [1,2]. Consequently, mitigating damage and stress is crucial for enhancing farmed animals’ survival rate and welfare [3]. Anesthetic use is a practical approach to reducing animal injury and stress resulting from human operations.

Anesthetics are broadly categorized into synthetic and natural types [4]. Ethanol, MS-222, and magnesium chloride are commonly used synthetic anesthetics in aquaculture research. Clove oil, a primary natural anesthetic in aquaculture, is renowned for its effectiveness, low risk of poisoning, antibacterial properties, and antioxidant effects [5]. Clove oil and its derivatives as anesthetic agents for decapod crustaceans have been investigated in other crustaceans for many years [6]. MS-222 is one of the most widely used anesthetics for poikilotherms worldwide [7]. Some studies have reported the anesthetic effect of MS-222 on crustaceans such as blue crabs (*Callinectes sapidus*), red swamp crayfish (*Procambarus clarkii*), and gammarids (*Gammarus pulex*) [8,9]. Ethanol, as an anesthetic in the neurotransmitter signaling pathway, could bind to specific receptors and promote the opening of ion channels, which leads to changes in the content of neurotransmitters, and it achieved anesthesia in crustaceans such as pacific white shrimp (*Litopenaeus vannamei*) and river prawn (*Macrobrachium tenellum*) [10,11,12]. Magnesium chloride was used in adult crayfish (*Astacus astacus*, *and Astacus leptodactilus*) and juvenile lobsters of European lobsters (*Homarus gammarus*) to investigate neural responses during stunning and killing [13]. The ideal properties of anesthetics are chemically stable, safe, effective, and without side effects [14]. Various factors influence their effectiveness and safety. These factors include abiotic elements such as temperature and salinity and biological factors like species, growth stage, and weight. For instance, transportation at high temperatures using magnesium chloride led to increased mortality in sea urchins (*Paracentrotus lividus*) [15]. Clove oil was only partially effective at the concentration of 20 mL L^−1^, and MS-222 had no anesthetic effects on the Chinese mitten crab (*Eriocheir sinensis*) [16]. Thus, exploring the anesthetic effects of different anesthesia treatments with specific species under various conditions is necessary.

Assessing the efficacy of anesthesia involves observing behaviors such as active movement and aggression [17]. Clove oil, for example, induced struggling in cuttlefish but limited their control over arm movement, while magnesium chloride caused erratic behavior [18]. Behavioral analysis software has also been employed to quantitatively analyze movement characteristics in abalone, providing insights into the effects of environmental factors [19]. Moreover, metabolomics, particularly NMR-based metabolomics, is a powerful tool to identify metabolite changes influenced by environmental factors [20]. It has been used to analyze many small molecules involved in physiological and pathological processes, such as the metabolic response of swimming crab (*Portunus trituberculatus*) under dibutyl phthalate pollution [21]. In this study, NMR-based metabolomics analysis was applied to investigate metabolite changes in the nervous system of mud crabs under the influence of clove oil anesthesia.

The mud crab, a significant economic marine species, is widely distributed in the Indo-West-Pacific region, with *S. paramamosain* being the predominant aquaculture species in China [22]. Despite advancements in mud crab breeding technology, reliance on wild seedlings remains due to specific challenges in artificial breeding technology. Modern genetic breeding necessitates the collection and analysis of parental phenotype data. With its non-invasive and non-direct contact advantages, computer vision technology has been widely adopted for this purpose. However, the unpredictable nature of mud crab activity poses challenges, making anesthesia a valuable tool for improving data accuracy and efficiency while minimizing animal stress.

While anesthetics are routinely employed in scientific research on crustaceans and the methods to induce analgesia and anesthesia were recently reviewed [3], the field of mud crab anesthesia remains underexplored. This study sought to address this gap by evaluating the efficacy of clove oil, MS-222, ethanol, and magnesium chloride. The assessment, based on parameters such as anesthesia duration, recovery time, 24-hour survival rate, and observed behavior, provides valuable insights into the optimal use of anesthetics in mud crab aquaculture and holds significant implications for their cultivation and circulation. These findings contribute to the broader understanding of mud crab welfare and may inform practices that enhance the overall success of mud crab farming.

## 2. Materials and Methods

### 2.1. Experimental Animal and Rearing Conditions

The mud crabs used in the present study were obtained from Jiashun Aquatic Cooperative (Taizhou City, China). The crabs were transferred to the pilot base at Ningbo University Meishan Campus. Before the experiment, crabs were acclimatized for seven days in the canvas pools with 0.6 m^3^ of water. During the acclimation, crabs were fed a commercial diet at 20:00, and 10% of the water was changed daily (8:00 a.m.). In this process, the salinity ranged from 23 to 25 ppt, dissolved oxygen (DO) was >8 mg L^−1^, water temperature was 22–24 °C, and the total ammonia nitrogen (TAN) and NO_2_-N was <0.5 mg L^−1^. After acclimation, the 520 healthy and intact crabs were randomly selected and starved for 24 hours before the experiments. 

### 2.2. Anesthetic Agents

Clove oil (98.5% Eugenol), ethanol (100%), magnesium chloride (MgCl_2_), and MS-222 were purchased from Sinopharm Chemical Reagent Company Limited (Shanghai City, China). The clove oil was initially diluted in ethanol with a 1:9 ratio to prepare a stock solution [23]. In addition, magnesium chloride was prepared with distilled water to avoid the formation of precipitation [24]. MS-222 and ethanol were added directly to seawater to the desired test concentration.

### 2.3. Criteria and Definitions

The criteria for determining the efficacy of different anesthetics were based on the behavioral responses [25]. Detailed information about the stages of anesthesia and recovery-associated behaviors in mud crabs is listed in Table 1. The mud crabs were exposed to different concentrations (details in Section 2.4) of clove oil, MS-222, ethanol, and magnesium chloride. The details of anesthesia time, recovery time, and behavioral animal trajectory tracking system (EthoVision XT, https://www.noldus.com/ethovision-xt, accessed on 10 December 2023) analysis indicators involved in the experiment are shown in Table S1.

### 2.4. Experimental Design and Sampling

#### 2.4.1. Discovery of Optimal Anesthetics

As established in previous studies, the criteria for optimal anesthesia necessitate the aquatic animals to be anesthetized within 3 min and recover within 5 min [26]. Therefore, 160 mud crabs (2.41 ± 0.65 g) were randomly selected after 24 hours of starvation. Separately, 8 mud crabs were exposed to different concentrations of clove oil (1.2, 2.4, 4.8, 7.2, 9.6 mg L^−1^), MS-222 (1, 2, 3, 4, 5 g L^−1^), ethanol (50, 100, 200, 300, 400 mg L^−1^), and magnesium chloride (200, 300, 400, 500, 600 g L^−1^). Each treatment involved 8 mud crabs, and individual observations were conducted throughout the experimental process. Specifically, the crabs were immersed in the respective anesthetic solutions, and after the anesthesia induction test, they were promptly transferred to a recovery device containing seawater without anesthetic. Anesthesia and recovery times were recorded during this process. After recovery, the mud crabs were placed in a temporary sample device, and mortality was monitored for 24 hours. Subsequently, 80 mud crabs (17.31 ± 3.98 g) were exposed to clove oil (2, 4, 6, 8, 10 mg L^−1^) and ethanol (50, 100, 200, 300, 400 mg L^−1^). To further screen the optimal anesthetic, each treatment was composed of 8 mud crabs with digital video cameras to compare the behavioral response.

All crabs, both males and females, were randomly selected and subjected to a 24-hour period of starvation before the commencement of the experiment. Subsequently, the crabs were captured and immersed in 1000 mL of water containing an anesthetic solution. Upon reaching anesthetic stage II, the crabs were promptly removed and transferred to 1000 mL of seawater without any anesthetic. The aquaria used for both anesthesia and recovery had dimensions of 20 cm × 20 cm × 20 cm. It is worth noting that the size and volume of the aquaria remained consistent across all experiments.

#### 2.4.2. Effects of Optimal Anesthetic on Physiology and Metabolism of Mud Crabs and Efficacy

To assess the impact of clove oil concentration on mud crabs of varying sizes, 280 mud crabs (0.03 ± 0.01 g) were exposed to different levels of clove oil (0.2, 0.4, 0.8, 1.5, 3.0 mg L^−1^), and mud crabs with higher body weights (7.04 ± 1.54 g, 15.10 ± 2.25 g, 27.50 ± 3.26 g, 104.83 ± 12.56 g, 159.23 ± 37.66 g, 399.73 ± 56.36 g) were exposed to clove oil concentrations of 2, 4, 6, 8, and 10 mg L^−1^, respectively. Eight mud crabs were observed in each treatment, and anesthesia and recovery times were recorded. After recovery, the crabs were temporarily relocated to a prepared sample device, with mortality monitored for 24 hours. After confirming clove oil’s anesthetic efficacy, its effects on the serum physiology, biochemistry, and antioxidant capacity of mud crab hepatopancreas were examined. Mud crabs (7.04 ± 1.54 g) were exposed to the optimal clove oil concentration (8 mg L^−1^). In this study, the hemolymph and hepatopancreas samples were collected from crabs before anesthesia (CG), after 3 min of anesthesia with clove oil (AG), upon initial return to the normal active stage (RG), and at 3 h (R3G) and 6 h (R6G), and 6 crabs were sampled at each time point.

Hemolymph was extracted from the base of the swimmeret of mud crabs, transferred to tubes containing an anti-coagulant (heparin), and centrifuged at 4 °C to obtain serum. The serum was stored at −80 °C for the analysis of cortisol, glucose, lactate concentration, aspartate aminotransferase (AST), and alanine aminotransferase (ALT). The hepatopancreas was quickly dissected, frozen in liquid nitrogen, and stored at −80 °C for hepatic superoxide dismutase (SOD), catalase (CAT), and glutathione peroxidase (GSH-Px) activity and malondialdehyde (MDA) content analysis.

For ^1^H NMR-based metabolomic analysis, the effects of clove oil on the metabolic phenotype of the thoracic ganglion tissue of mud crabs were investigated. Mud crabs (399.73 ± 56.36 g) were exposed to optimal concentrations of clove oil (20 mg L^−1^). In this study, the thoracic ganglia samples were collected from crabs before anesthesia (CG), after 3 min of anesthesia with clove oil (AG), and upon returning to complete recovery (RG), and 6 crabs were sampled at each time point. The mud crabs were placed on ice, and thoracic ganglia were removed, immediately snap-frozen in liquid nitrogen, and stored at −80 °C for ^1^H NMR analysis.

### 2.5. Analysis of Serum Biochemical and Hepatic Antioxidant Capacity

Six hemolymph samples in each replicate from each treatment were selected for physiological and biochemical analysis. Hemolymph cortisol levels were determined by an ELISA kit (Enzyme-linked Biotechnology, Shanghai, China). The assay was carried out on microplates based on the principle of competitive binding. Firstly, samples, standards, and horseradish peroxidase-labeled detection antibodies were added to the microplate in sequence. Then, the microplate was incubated at 37 °C for 60 min and thoroughly washed with detergent. The substrate for color development, tetramethylbenzidine (TMB), changed from blue to yellow, and the yellow depth of the reaction’s final solution was proportional to the measured cortisol content. The absorbance at 450 nm was read within 15 min using an Absorbance Microplate Reader (Spectra Max 190, Molecular Device, Sunnyvale City, CA, USA) [27]. The glucose (A154-1-1), lactate (A019-2-1) levels and AST (C00-1-1) and ALT (C010-2-1) activities in the hemolymph were determined using commercially available kits (Nanjing Jiancheng Bioengineering Institute, Nanjing, China).

Six hepatopancreas samples in each replicate from each treatment were selected for antioxidant capacity analysis. Before antioxidant capacity analysis, samples were homogenized in ice-cold normal saline and centrifuged at 825× *g* at 4 °C for 10 min. The SOD (A001-3-2), CAT (A007-1-1), and GSH-Px (A006-2-1) activity and the MDA (A003-1-2) content were measured using commercial detection kits (Nanjing Jiancheng Bioengineering Institute, Nanjing, China). The operation steps were according to the corresponding commercial kits’ instructions.

### 2.6. NMR-Based Metabolomic Analysis

Each of the nervous systems (about 0.1 g) from 18 crab samples were added into a 2.5 mL Eppendorf tube with crushing beads and 1 mL aqueous methanol (methanol/water = 2:1). The supernatants of each sample were collected after homogenization and centrifugation. After repeating the above extraction, the resultant two supernatants were combined and freeze-dried in a vacuum pump after removing the methanol. The extracts of each sample were then dissolved separately in 600 μL phosphate buffer. Following centrifugation, 550 μL of the supernatant from each extract was moved into a 5 mm NMR tube for NMR analysis. NMR spectroscopic analysis was described previously [28]. For each of ^1^H NMR spectra, the region of δ 9.3–0.7 without residual water signal of δ 5.2–4.7 and methanol signal of δ 3.38–3.34 was binned with an equal width of 0.004 ppm (2.4 Hz). To compensate for overall concentration differences, all bins of NMR data were averaged and normalized to the wet weight of the corresponding sample. Before the multivariable data analysis, these binned data were averaged and normalized to the wet weight of the corresponding sample to compensate for overall concentration differences.

The normalized NMR data were imported into SIMCA-P^+^ (version 12.0, Umetrics, Umeå, Sweden) for multivariate data analysis. The normalized NMR data were used for principal component analysis (PCA). NMR data were automatically normalized for orthogonal partial to latent structure discriminant analysis (OPLS-DA) with NMR data as X and group information as Y matrixes. The reliability of the OPLS-DA model was verified by 9-fold cross-validation [29]. The higher the Q^2^ value of the model quality parameter, the more reliable the quality of the model. The reliability of the OPLS-DA model required further evaluation, with cross-validation of the analysis of variance approach, and only OPLS-DA models with *p* < 0.05 were reliable. To obtain metabolites that contribute significantly to the discrimination between the two groups, the loadings of the NMR data were back-scaled transformed and automatically normalized [30]. The correlation coefficient graph of the OPLS-DA model was drawn using MATLAB software (V 7.10). The color of each point on the correlation coefficient plot indicates the NMR data weight size, and the weight represents Pearson’s product-moment correlation coefficient (r) value between the X and Y variables. The absolute value of the correlation coefficient (|R|) was used in this study, and each metabolite with |r| > 0.522 was considered statistically significant (*p* < 0.05) [31,32].

### 2.7. Statistical Analysis

All data were expressed as mean ± standard deviation. The data do not conform to the analysis of variance. The results were tested via the Kruskal–Wallis test using SPSS20.0 software. Differences were considered statistically significant at *p* < 0.05. Prism 8 software (version 8.01, GraphPad Software Inc., La Jolla, CA, USA) was used for data visualization.

## 3. Results

### 3.1. Anesthesia and Recovery Times for Four Anesthetics

**Figure 1:**
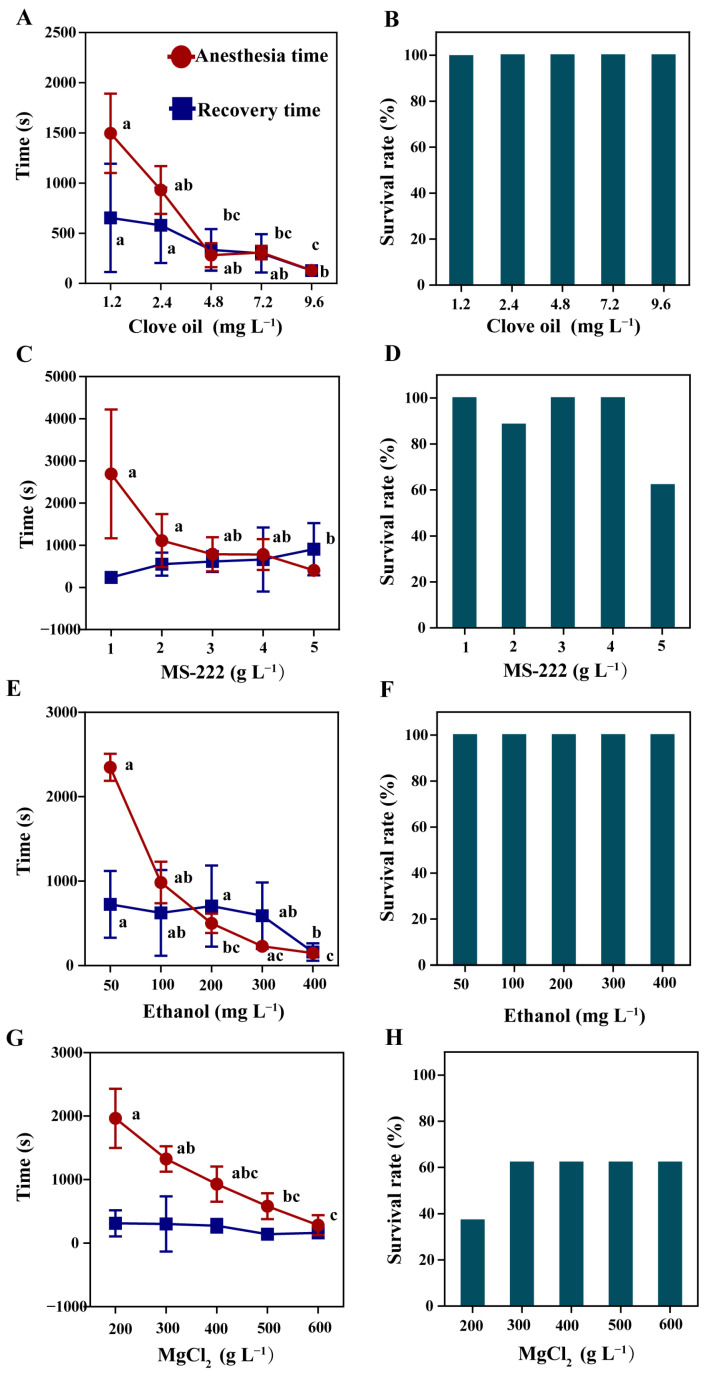
Anesthesia and recovery time of mud crab with clove oil (**A**), MS-222 (**C**), ethanol (**E**), and magnesium chloride (MgCl_2_) (**G**). 24-hour survival rate after anesthesia with clove oil (**B**), MS-222 (**D**), ethanol (**F**), and magnesium chloride (**H**). Time is referred to as seconds. Data are presented as mean ± SD. There were statistically significant differences in anesthesia timelines and recovery timelines plots with different lowercase letters (*p* < 0.05, *n* = 8). The same applies below.

The anesthetic effects of clove oil, MS-222, ethanol, and magnesium chloride on the mud crabs are presented in Figure 1. After exposure to clove oil, the anesthesia time for mud crabs decreased with the increasing anesthetic concentration (Figure 1A, *p* < 0.05). There was no significant difference in recovery time between anesthetic concentrations from 1.2 to 7.2 mg L^−1^ (Figure 1A, *p* > 0.05), and the recovery time decreased significantly in the 9.6 mg L^−1^ group (Figure 1A, *p* < 0.05). Moreover, the 24-hour survival rate of crabs under clove oil treatment was 100% (Figure 1B). Similarly, with the MS-222, ethanol, and magnesium chloride treatment, the anesthesia time for mud crabs decreased with the increase in anesthetic concentration (Figure 1C,E,G, *p* < 0.05). The anesthesia time for 5 g L^−1^ MS-222 was significantly shorter than that for 1 g L^−1^ and 2 g L^−1^ MS-222 (Figure 1C, *p* < 0.05). The anesthesia time for 400 mg L^−1^ ethanol was significantly shorter than that for 50 mg L^−1^ and 100 mg L^−1^ ethanol (Figure 1E, *p* < 0.05). The anesthesia time for 600 g L^−1^ magnesium chloride was significantly shorter than that for 200 g L^−1^ and 300 g L^−1^ magnesium chloride (Figure 1G, *p* < 0.05). The recovery time was not affected by the concentration of MS-222 and magnesium chloride (Figure 1C,G, *p* > 0.05). The recovery time for 400 mg L^−1^ ethanol was significantly shorter than that for 50 mg L^−1^ and 200 mg L^−1^ ethanol (Figure 1E, *p* < 0.05). The mortality was found when crabs were treated with MS-222 and magnesium chloride (Figure 1D,H); however, the 24-hour survival rate of mud crabs exposed to ethanol solutions was 100% (Figure 1F). 

### 3.2. Effects of Clove Oil and Ethanol Concentration on the Behavior of Mud Crab

**Figure 2:**
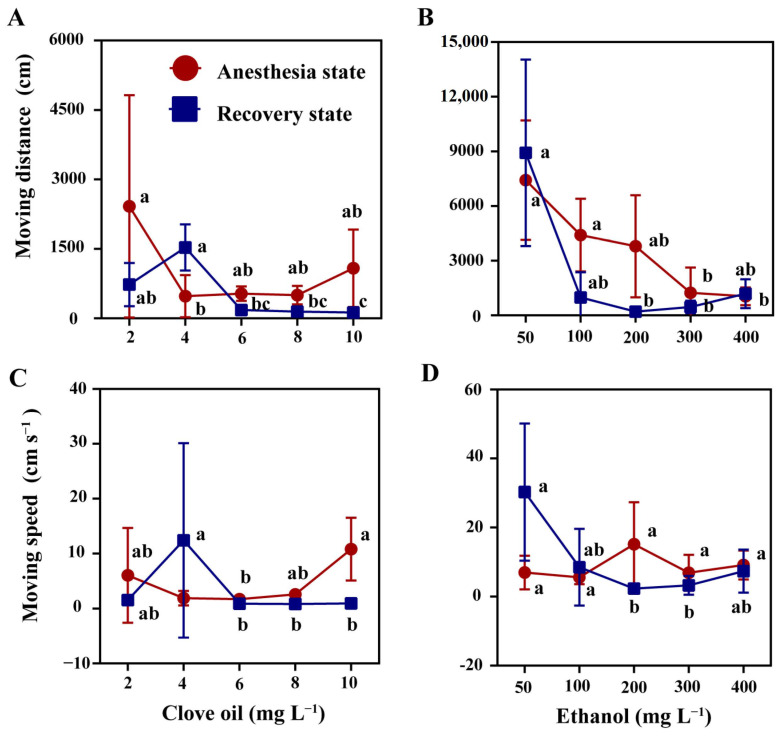
The behavior of the mud crab during anesthesia and recovery. Moving distance and moving speed of mud crab during anesthesia and recovery state with clove oil (**A**,**C**) and ethanol (**B**,**D**). There were statistically significant differences in anesthesia timelines and recovery timelines plots with different lowercase letters (*p* < 0.05, *n* = 8).

**Figure 3:**
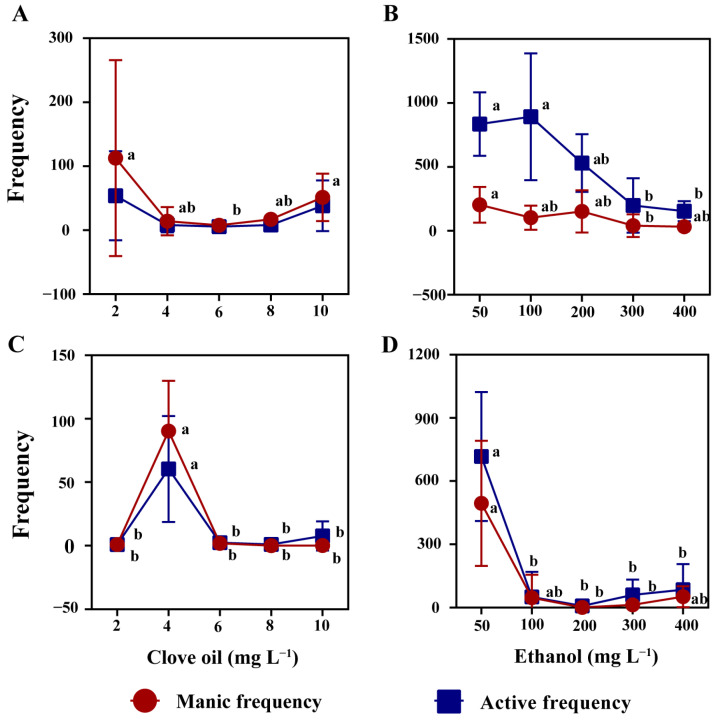
Manic and active behavior frequency of mud crab during anesthesia and recovery. Clove oil and ethanol anesthesia state (**A**,**B**). Clove oil and ethanol recovery state (**C**,**D**). There were statistically significant differences in anesthesia timelines and recovery timelines plots with different lowercase letters (*p* < 0.05, *n* = 8).

An increase in the concentration of clove oil and ethanol resulted in a significant decrease in the moving distance of mud crabs during the anesthesia stage (Figure 2A,B, *p* < 0.05). Similarly, a decrease in the moving speed of mud crabs was observed during the recovery stage (Figure 2C,D, *p* < 0.05). Notably, the moving distance and moving speed of mud crabs anesthetized by clove oil was significantly higher than that of ethanol (Figure 2). At the same time, it was found that the manic and active behavior frequency of mud crabs was more intense with the ethanol solutions (Figure 3), and the phenomenon of amputation occurring in mud crabs was observed.

### 3.3. Effect of Clove Oil Concentration on Mud Crab of Different Body Weight

**Figure 4:**
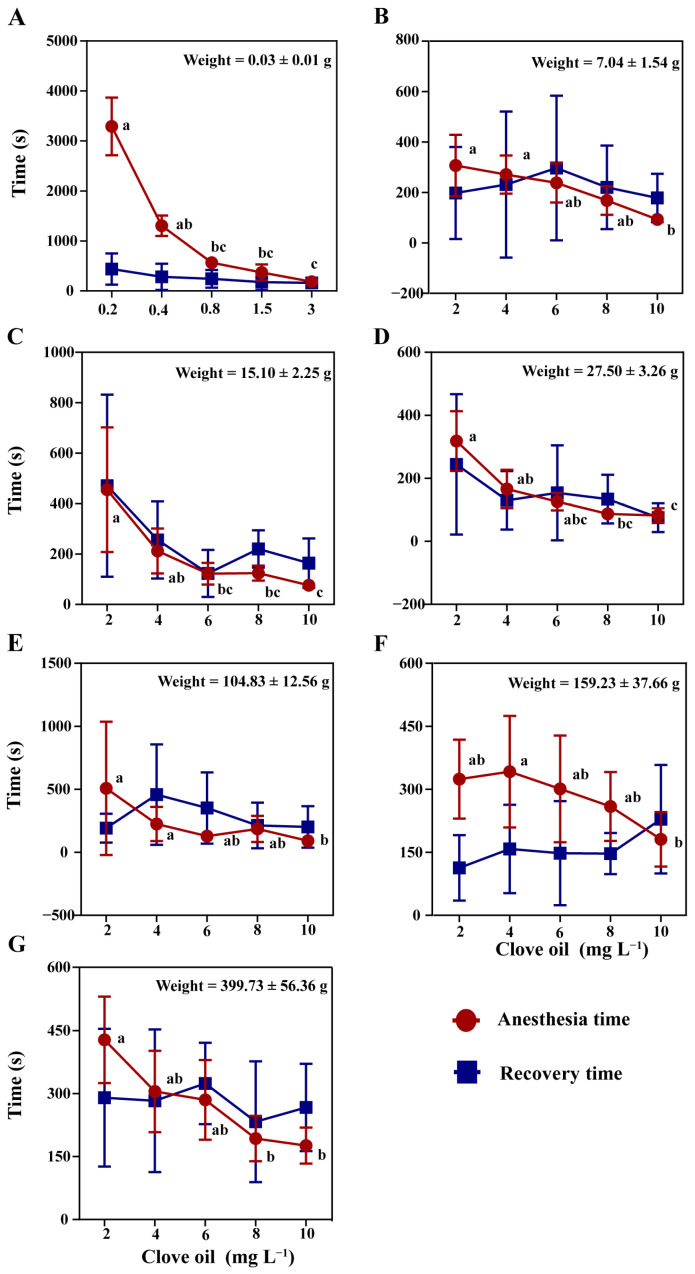
Anesthesia and recovery time of clove oil for mud crabs of different weights (**A**–**G**). There were statistically significant differences in anesthesia timelines and recovery timelines plots with different lowercase letters (*p* < 0.05, *n* = 8).

**Figure 5:**
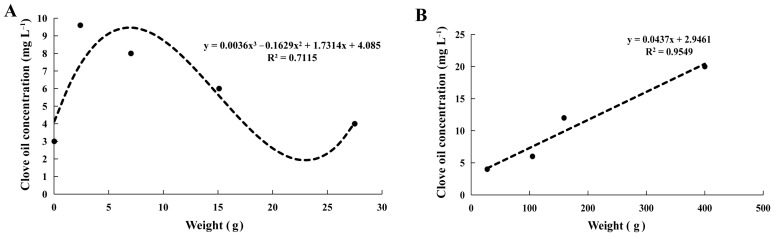
Cubic function relationship between 0.03–27.50 g body weight and clove oil (**A**), and 27.50–399.73 g body weight and clove oil primary function relationship (**B**). The black dots represent the weight of the crabs involved in the experiment.

The effect of clove oil concentrations on mud crabs of varying sizes was evaluated. The results showed that the anesthesia time for crabs decreased with the increase in anesthetic concentrations (Figure 4A–G, *p* < 0.05) with 100% 24-hour survival rates in all groups. The recovery time was not affected by body weight and the concentrations of clove oil (Figure 4A–G, *p* > 0.05). They were found that the optimal anesthetic concentrations for 0.03 ± 0.01 g, 7.04 ± 1.54 g, 15.10 ± 2.25 g, 15.10 ± 2.25 g, 104.83 ± 12.56 g, 159.23 ± 37.66 g, and 399.73 ± 56.36 g mud crabs were 3 mg L^−1^, 8 mg L^−1^, 6 mg L^−1^, 4 mg L^−1^, 6 mg L^−1^,12 mg L^−1^, and 20 mg L^−1^, respectively. Through regression analysis, the correlation between the optimal anesthetic concentration of clove oil depends on the weight of the crab, for crabs weighted from 0.03 to 27.50 g, the cubic function relationship was y = 0.0036 x^3^ − 0.1629 x^2^ + 1.7314 x + 4.085 (R^2^ = 0.7115); for those weighted from 27.50 to 399.73 g, the primary function relationship was y = 0.0437 x + 2.9461 (R^2^ = 0.9549) (Figure 5).

### 3.4. Hemolymph Biochemical Parameters and Hepatopancreas Antioxidant Capabilities with Clove Oil Treatment

**Figure 6:**
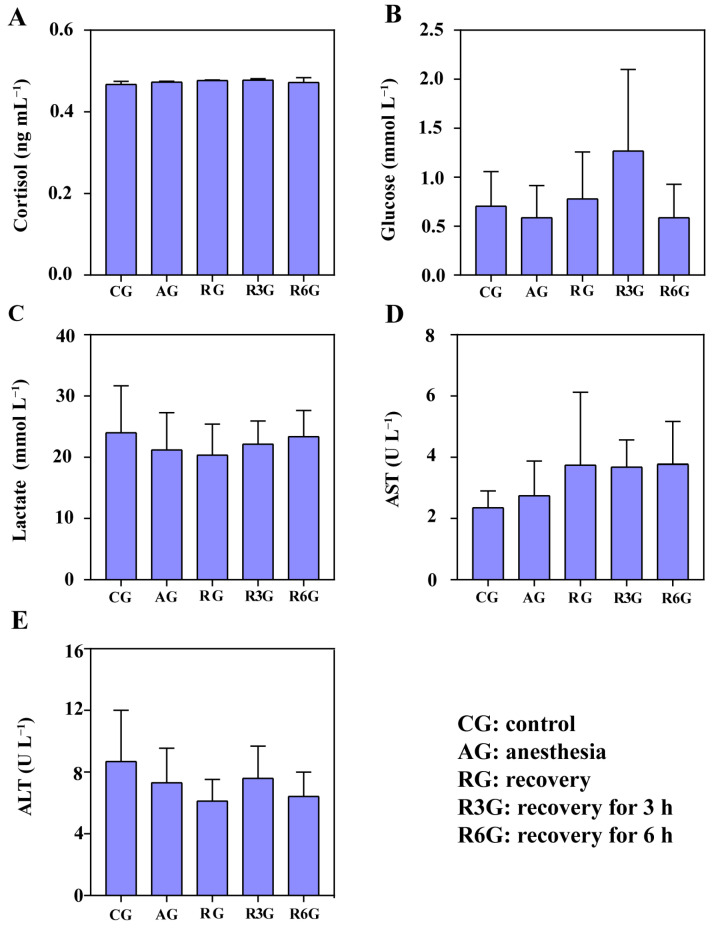
The changes in hemolymph cortisol (**A**), glucose (**B**), lactate (**C**), ALT activity (**D**), and AST activity (**E**) in mud crab during anesthesia (AG) and recovery (RG, R3G, R6G) with clove oil. There are statistical differences in bar charts with different superscripts (*p* < 0.05, *n* = 6). Same below.

**Figure 7:**
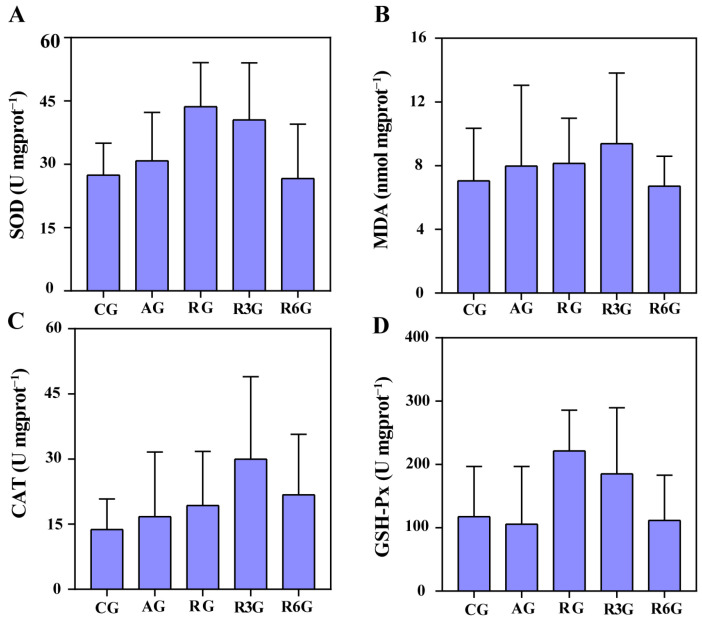
Hepatopancreas SOD (**A**), MDA (**B**), CAT (**C**), and GSH-Px (**D**) levels of mud crab anesthetized with clove oil.

Under the effect of clove oil, the contents of hemolymph glucose, cortisol, and lactate of crabs were not significantly different at AG, RG, R3G, and R6G stages in each treatment group (Figure 6A–C, *p* > 0.05). There was no significant difference in serum ALT and AST activity at AG, RG, R3G, and R6G stages in each treatment (Figure 6D,E, *p* > 0.05). There were no significant changes in MDA, SOD, CAT, and GSH-Px in the hepatopancreas of mud crabs in each treatment group (Figure 7A–D, *p* > 0.05).

### 3.5. Changes in Metabolites in Crabs Treated with Clove Oil

**Figure 8:**
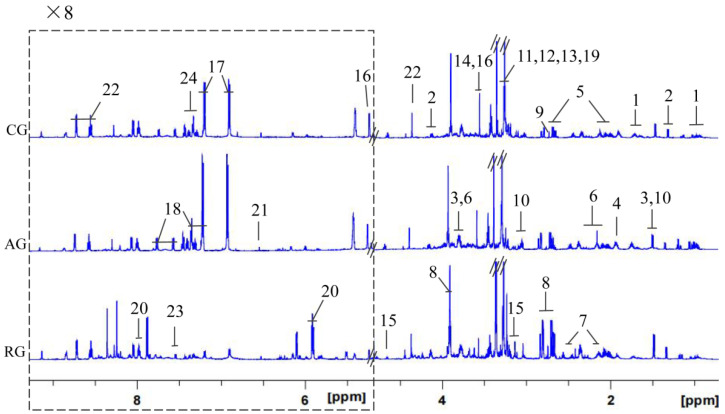
Typical 600 MHz ^1^H NMR spectrum of nervous system extracts from mud crab. The spectral region δ 5.2–9.3 is displayed at 8-fold magnification compared to the region δ 0.7–4.8. CG: control group; AG: anesthesia group; RG: recovery group.

**Figure 9:**
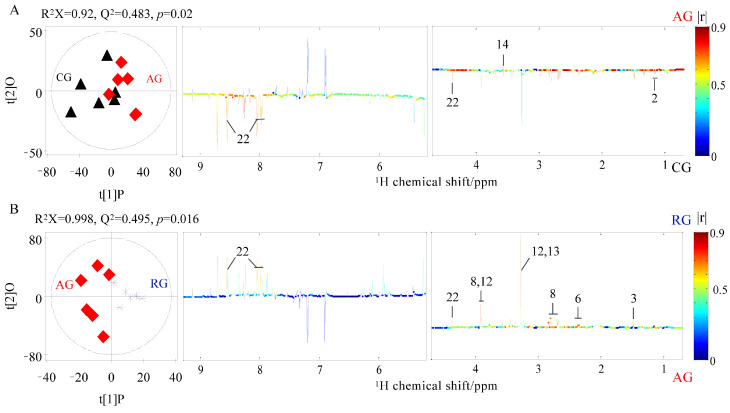
OPLS-DA score plot (**left**) and corresponding color-coded correlation coefficient plot (**right**) obtained by comparisons between nervous system extract spectra of mud crabs in control group CG (black triangle) and anesthesia treatment group (red oblique square) (**A**), and anesthesia treatment group and recovery group (blue snowflake) (**B**).

The representative 600 MHz ^1^ H NMR spectra of the nervous system extracts obtained from mud crab (including the control and clove oil treatment group) were shown in Figure 8 and the ^1^H-^1^H correlation was provided by two-dimensional spectrum. A total of 24 metabolites were identified in the ^1^ H NMR spectra of the nervous system and were classified in Table 2. The OPLS-DA model further distinguished the metabolic changes in mud crabs treated with clove oil. The results showed that glycine content in the AG group was significantly increased compared with the CG group, and the contents of lactate and 2-pyridinemethanol decreased significantly (Figure 9A, *p* < 0.05). However, no significant changes in metabolites were observed in the RG group compared to the CG group (*p* > 0.05), thus precluding the establishment of an OPLS-DA model for both groups. The contents of the metabolites such as glutamate, alanine, aspartic acid, betaine, taurine, and 2-pyridinemethanol in the RG group were significantly higher than in the AG group (Figure 9B, *p* < 0.05). The correlation coefficients of the significantly altered metabolites are summarized in Table 3.

## 4. Discussion

Anesthetics are crucial in inducing sedation in aquatic organisms and reducing operational stress. However, to date, no studies have investigated the effectiveness of anesthetics in mud crabs. Currently, clove oil, MS-222, ethanol, and magnesium chloride have been employed in crustaceans such as blue crabs, crayfish, gammarids, pacific white shrimp, and European lobsters [3,8,9,25]. Nevertheless, lower survival rates were detected in crabs exposed to MS-222 and magnesium chloride compared with clove oil and ethanol in this study. Consequently, MS-222 and magnesium chloride anesthetics were excluded as effective options for mud crabs.

As a complement to physiology, behavior is a valuable tool for evaluating the adaptation of animals to an aquaculture environment [33,34]. Behavioral parameters, such as moving distance and speed, are effective indicators for evaluating stress levels. Previous studies have found that exposure to blue light induces stress in abalone, leading to increased moving speed and distance, likely as an attempt to find a hiding place [35]. For fish, a high concentration of anesthetics increases moving distance and speed due to stimulation of the skin, gills, and taste-smell receptors [36]. In the present study, we found that crabs in ethanol anesthesia had higher moving distance and speed and manic and active behavior frequency than in clove oil, accompanied by limb amputation, indicating a higher stress level. Therefore, it could be preliminarily judged that clove oil is an optimal anesthetic for mud crabs.

Mud crabs, a species that are highly tolerant to air exposure, and mud crabs with different weights could be found in the market; thus, in the present study, we further assessed the dose–response relationship between clove oil and mud crabs of different body weights. The correlation between the optimal anesthetic concentration of clove oil depends on the weight of the crab. Our results show that there is a cubic function relationship for crabs weighing less than 27.50 g and a primary function relationship for crabs weighing between 27.50 g and 399.73 g. These findings indicated that the optimal anesthetic concentration of clove oil is highly dependent on body weight.

Anesthetics are often stressors, stimulating cortisol release in aquatic animals [37,38]. Fish, during anesthesia, may experience short-term hypoxia due to inadequate ventilation, leading to increased cortisol levels [39]. Cortisol, in turn, activates gluconeogenesis and glycogenolysis, increasing blood glucose production to meet elevated energy demands. Additionally, hypoxia results in anaerobic glucose metabolism and lactate formation [40]. As a result, blood cortisol, glucose, and lactate levels are commonly used as valuable parameters in aquatic biological anesthesia studies. The findings of this study indicate that the optimal concentration of clove oil had no significant effect on hemolymph cortisol, glucose, and lactate contents in mud crabs during both the anesthesia and recovery stages. In contrast, a separate study found that the glucose of freshwater prawns (*Macrobrachium rosenbergii*) was higher when exposed to 30 min with eugenol [41]. Another study found that clove oil significantly increased the cortisol, glucose, lactate, hemoglobin, and hematocrit levels of the spotted knifejaw, but these parameters returned to near-normal levels after 6 h [42].

Alanine transaminase (ALT) and aspartate transaminase (AST) are ubiquitous transaminases released into the circulation after tissue damage and dysfunction, reflecting the degree of liver damage in aquatic animals [43]. The ALT activity of freshwater prawns was higher when exposed to eugenol for 30 min [41]. In the present study, the optimal concentration of clove oil had no significant effect on hemolymph ALT and AST activity in mud crabs during both the anesthesia and recovery stages. Therefore, the results from the present study suggest that there is no stress response in mud crabs (7.04 ± 1.54 g) under the optimal concentration of clove oil, and using clove oil as an anesthetic for mud crabs is highly safe.

The hepatopancreas, a crucial metabolic and immune organ, exhibits a high antioxidant defense capacity, and its stress resistance is intricately regulated by enzymes, hormones, and neuronal signals [42]. Superoxide dismutase (SOD), catalase (CAT), and glutathione peroxidase (GSH-Px) are key members of antioxidant enzymes, playing essential roles in balancing oxidative stress products, while malondialdehyde (MDA) is a byproduct of lipid peroxidation reactions [27]. High-concentration anesthetics, as stress sources, can induce oxidative stress in the hepatopancreas and alter the antioxidant state of aquatic organisms [44]. For example, the CAT activity in the white shrimp treated with eugenol did not differ from that of the other treatments [45]. Another study found that eugenol anesthesia in Nile tilapia can lead to significant and transient increases in GSH and CAT levels during anesthesia, which are entirely reversed during recovery. However, MDA and SOD do not show significant changes at all stages on average [38]. In our study, no significant changes were found in the activities of antioxidant enzymes and the content of MDA in the hepatopancreas of mud crabs before and after anesthesia with clove oil. Therefore, clove oil does not induce oxidative damage in mud crabs.

The mechanism of anesthetics could be determined through neurotransmitter changes [18]. Neurotransmitters, chemical messengers released by neurons, mediate intercellular communication, regulating central and peripheral physiological functions in the nervous system [46]. The central nervous system of the mud crab is composed of a brain and ventral nerve cord, which are similar to other decapods [47]. In our study, non-targeted NMR-based metabolomic analysis indicated a significant increase in inhibitory neurotransmitter glycine in the anesthesia group compared to the recovery group. Glycine binds to receptors, stimulating the transmembrane flow of chloride ions, preventing depolarization and neuronal discharge induced by excitatory neurotransmitters, and causing neuronal hyperpolarization, thereby reducing neuronal excitability [48]. This may explain why clove oil exerts its anesthetic effect on mud crabs. Additionally, glutamic acid and aspartic acid in the recovery group significantly increased [49,50]. These excitatory neurotransmitters in the central nervous system potentially contribute to the recovery of mud crabs after anesthesia. Importantly, excitatory and inhibitory neurotransmitters recovered to normal levels after anesthesia, suggesting that clove oil does not negatively affect the neurotransmitters of mud crabs. 

## 5. Conclusions

In summary, our comprehensive research, encompassing behavioral, physiological, and metabolomic analyses, unequivocally establishes clove oil as the optimal anesthetic for mud crabs. The mechanism underlying its efficacy involves the modulation of excitatory and inhibitory neurotransmitter levels. Furthermore, our investigation reveals a clear correlation between the optimal anesthetic concentration of clove oil and the weight of mud crabs. These equations underscore the robust relationship between the anesthetic concentration and the weight of mud crabs. In conclusion, clove oil emerges as a safe and optimal anesthetic agent for mud crabs, offering valuable insights for research and practical aquaculture applications.

## Figures and Tables

**Table 1:** Standard of anesthesia and recovery-associated behaviors.

Behaviors/Response	
Stages of anesthesia	
I	Crabs partially lose their balance and righting reflexes, and their response to stimuli persists
II	Crabs lose balance and righting reflexes, respond weakly to stimuli, do not retract their limbs, and lose their defensive behavior
III	Unconscious, immobile, unresponsive to stimuli
IV	Excessive anesthesia leads to crab death
Stages of recovery	
I	Partial regain of equilibrium, response to external stimuli
II	Crabs have defensive behavior respond quickly to stimulation, and resume feeding behavior

**Table 2:** NMR data of metabolites detected in the nervous system extracts.

Key	Metabolites	Moieties	δ^1^ H (ppm) and Multiplicity ^a^
1	Leucine	aβCH_2_, γCH, δCH_3_, δ’CH_3_	1.73(m), 1.66(m), 0.98(d), 0.96(d)
2	Lactate	αCH, βCH_3_, COOH	4.13(q), 1.33(d)
3	Alanine	αCH, βCH_3_, COOH	3.77(q), 1.48(d)
4	Acetate	CH_3_, COOH	1.92(s)
5	Methionine	βCH_2_, γCH_2_, S-CH_3_	2.15(m), 2.65(t), 2.14(s)
6	Glutamate	δCO, αCH, βCH_2_, γCH_2_, COOH	3.78(m), 2.12(m), 2.05(m), 2.35(dt)
7	Glutamine	αCH, βCH_2_, γCH_2_	3.77(t), 2.14 (m), 2.46(m)
8	Aspartate	αCH, βCH_2_, γCOOH	3.90(dd), 2.83(dd), 2.68(dd)
9	Sarcosine	CH_2_, N-CH_3_, COOH	3.61(s), 2.74(s)
10	Lysine	βCH_2_, γCH_2_, δCH_2_, εCH_2_	1.92(m), 1.48(d), 1.73(m), 3.03(t)
11	Arginine	γCH_2_, δCH_2_, εC	1.72(m), 3.27(t)
12	Betaine	CH_3_, CH_2_, COO-	3.26(s), 3.90(s)
13	Taurine	CH_2_SO_3_, CH_2_NH_2_	3.26(t), 3.43(t)
14	Glycine	αCH_2_, COOH	3.56(s)
15	β-Glucose	C_1_H, C_2_H, C_3_H	4.65(d), 3.22(dd), 3.45(m)
16	α-Glucose	C_1_H, C_2_H, C_3_H	5.24(d), 3.54(dd), 3.72(m)
17	Tyrosine	RingC_2_, _6_H, RingC_3_,_5_H	7.21(d), 6.90 (d)
18	Tryptophan	RingC_2_H, RingC_3_, RingC_4_H, RingC_5_H, RingC_6_H, RingC_7_H, RingC_8_, RingC_9_	7.33(s), 7.55(d), 7.31(m), 7.20 (m), 7.74(d)
19	Trimethylamine-N-oxide	CH_3_	3.26(s)
20	Uridine	C_2_H, C_3_H, C_1′_H	7.99(d), 5.98(d), 5.97(m)
21	Fumarate	CH, COOH	6.52(s)
22	2-Pyridinemethanol	C_2_, C_3_H, C_4_H, C_5_H, C_6_H, C_7_H	8.71(dd), 7.97(td), 8.55(td), 8.04(dd), 4.37(s)
23	Uracil	C_4_H, C_5_H, C_1_, C_2_	7.54(d), 5.81(d)
24	Phenylalanine	Ring C_2_, _6_H, RingC_3_, _5_H, RingC_4_, RingC_1_	7.33(q), 7.43(t), 7.38(m)

^a^ Multiplicity: s, singlet; d, doublet; t, triplet; q, quartet; m, multiplet; dd, doublet of doublets; dt, doublet of triples; td, triples of doublet.

**Table 3:** The correlation coefficients (r) of significantly altered metabolites in the aqueous extracts of the nervous system of mud crabs after clove oil treatment.

Metabolites	Correlation Coefficients (r)	
	AG/CG	RG/AG
Lactate	−0.70	-
Alanine	-	0.56
Glutamate	-	0.67
Aspartate	-	0.71
Betaine	-	0.64
Taurine	-	0.60
Glycine	0.55	-
2-Pyridinemethanol	−0.66	0.65

The positive and negative signs of the correlation coefficients indicate the positive and negative correlation of the content, respectively. |r| = 0.522 was used as the corresponding cutoff value of the correlation coefficients for the statistical significance based on the discriminant significance. “-” means the correlation coefficient |r| is less than the cutoff value. CG, control group; AG, anesthesia group; RG, resuscitation group.

## Data Availability

The data presented in this study are available in review.

## Supplementary Materials

The following supporting information can be downloaded at: https://www.mdpi.com/article/10.3390/antiox12122124/s1, Table S1: The definition of indicators involved in the experiment.
