# Supplementary material for: Optimizing Anesthetic Practices for Mud Crab: A Comparative Study of Clove Oil, MS-222, Ethanol, and Magnesium Chloride
Source: Antioxidants (Basel). 2023 Dec 16;12(12):2124. doi: 10.3390/antiox12122124 (PMC10740467; doi:10.3390/antiox12122124)
Supplement: Supplementary file 1 [file antioxidants-12-02124-s001.zip › antioxidants-2762999-supplementary.pdf]

Table S1 The definition of indicators involved in the experiment

Definitions	Behavior description
Anesthesia time	The crab is put into the anesthetic solution until the crab reaches anesthesia stage II
Recovery time	After the crab reaches anesthesia state II，the crab is placed in seawater without anesthetic until the crab recovers as described in recovery stage I
Behavior analysis time in the anesthesia stage	The starting point of the video of behavior analysis in the anesthesia stage is when the mud crab is put into anesthesia and when the mud crab reaches the anesthesia stage II
Behavioral analysis time in the recovery stage	The beginning time of the video of behavioral analysis in the recovery stage is when the mud crab is put in clear water after reaching the anesthesia stage II, and the end time is when the mud crab responds to stimulation, which is the recovery stage I.
Manic state	Calculate the duration when it exceeds 60%, the animal is considered to be in a manic state
Active state	Calculate the duration when it falls within the range of 60-20%, it is deemed active state
Quiescent state	Calculate the duration when it drops below 20%, it enters a quiescent state
